# A Systematic Review on Functional Bioactive Compound Atractylone: Natural Source, Pharmacological Properties and Mechanisms Insights

**DOI:** 10.1002/fsn3.71488

**Published:** 2026-02-02

**Authors:** Hamza Elhrech, Oumayma Aguerd, Meriem El Fessikh, Zouhair Essahli, Taoufiq Benali, Waleed Al Abdulmonem, Learn‐Han Lee, Imane Chamkhi, Abdelhakim Bouyahya

**Affiliations:** ^1^ Laboratory of Human Pathologies Biology, Faculty of Sciences Mohammed V University in Rabat Rabat Morocco; ^2^ Laboratory of Ecotoxicology, Bioresources, and Coastal Geomorphology, Polydisciplinary Faculty of Safi Cadi Ayyad University Safi Morocco; ^3^ Department of Pathology, College of Medicine Qassim University Buraydah Saudi Arabia; ^4^ Novel Bacteria and Drug Discovery Research Group (NBDD), Microbiome Research Group, Research Centre for Life Science and Healthcare, Nottingham Ningbo China Beacons of Excellence Research and Innovation Institute (CBI) University of Nottingham Ningbo China Ningbo China; ^5^ Geobiodiversity and Natural Patrimony (GEOPAC), Scientific Institute Mohammed V University in Rabat Rabat Morocco

**Keywords:** anticancer, atractylone, inflammation, neuroprotective, pharmacokinetic, systematic review

## Abstract

Traditional Chinese medicine (TCM) has garnered considerable attention due to its multifaceted therapeutic potential, characterized by a broad spectrum of pharmacological activities, multiple biological targets, and a generally favorable safety profile. Atractylone, a bioactive sesquiterpenoid, has been noted to exhibit numerous pharmacological effects, including cytotoxic, antimicrobial, anti‐inflammatory, antiviral, anticancer, antioxidant, neuroprotective, and gastroprotective activities. The isolation and structural characterization of this compound are essential for optimizing its pharmacological applications and unlocking its full therapeutic potential. Despite its promising bioactivity, to our knowledge, no comprehensive review has yet been dedicated to summarizing the current state of research on Atractylone. To tackle this gap, we conducted a systematic review following the PRISMA guidelines to define a clear research question and methodology. A comprehensive literature search was performed using PubMed, Scopus, Web of Science, and Google Scholar to collect all available information on Atractylone. This review aims to furnish a detailed analysis of its natural sources, biosynthetic pathways, and biological activities. By summarizing current knowledge, this article establishes a foundation for future research and encourages further exploration of Atractylone's therapeutic applications.

## Graphical abstract

Atractylone exhibits diverse pharmacological effects, including anticancer and neuroprotection. A systematic review was conducted following PRISMA guidelines to analyze Atractylone. This study explores Atractylone's sources, biosynthesis, and therapeutic potential. The review establishes a foundation for future research on Atractylone's applications.

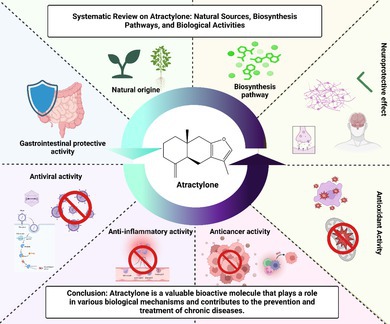

## 1 Introduction

Over the recent years, there has been an appreciation of natural products and their metabolites as a rich source of meaningful leads in the discovery and generation of novel pharmacological agents against a wide range of pathologies. The traditional Chinese medicine (TCM) has attracted much attention in the recent years due to the fact that it is a holistic form of treatment of diseases. TCM can take advantage of pharmacological multiple mechanisms, and can act simultaneously on different biological pathways instead of concentrating on one target. Another feature that is usually emphasized is its overall good safety results. TCM practitioners depend on medicinal plants, used singly or in mixtures, with the purpose of maximizing a good effect and getting a better clinical outcome. Within this context, *Atractylodes* species and several related plants have long been used in traditional practices, where their essential oils and extracts have demonstrated a broad range of biological effects.

Atractylone (Atr) is a sesquiterpene that is most frequently found in the Asteraceae family, with its richest source in the rhizomes of some species mainly from genus *Atractylodes* (Chen et al. 2016). The members of this family occur mostly in parts of China, Korea, Japan, India, and Thailand (Zhang, Wang, et al. 2023). Previous investigations have reported multiple pharmacological properties for Atr, including antiviral, anti‐inflammatory, anticancer, hepatoprotective, neuroprotective, and gastroprotective effects. These activities are supported by a growing number of in vitro and in vivo studies, suggesting that Atr may act through several interconnected molecular pathways. Despite this promising profile, research on Atr remains fragmented, and most available studies examine isolated biological activities without integrating its broader chemical and mechanistic context (Amorim et al. 2009; Chen et al. 2016; Cheng et al. 2016, 2019; Ren et al. 2021; Sun, Shi, et al. 2022; Sun, Luo, et al. 2022; Tian et al. 2019).

In experiments on SARS‐CoV‐2‐infected Vero E6 cells, it was demonstrated that Atr could lower the levels of several key cytokines (IL‐6, CCL‐2, TNF‐α, CCL‐3, and CXCL‐10) in a concentration‐dependent manner (Liu et al. 2024).

Pharmacokinetic data show that Atr can be absorbed in the intestine with relatively high efficiency, mainly through passive diffusion. This property suggests that oral delivery is a practical option. The permeability coefficients (Papp values) also support its effective intestinal absorption (Wu et al. 2009). In addition, its pharmacokinetic behavior points to good bioavailability. Atr is absorbed and distributed well, undergoes metabolism and elimination in a balanced way, and shows a half‐life that is considered suitable for therapeutic use.

A recent review by Yao et al. (2024) focused specifically on the antitumor activity of Atr, providing a useful overview of its effects within oncology (Yao et al. 2024). However, no work to date has offered a comprehensive synthesis that brings together its full pharmacological range, natural distribution, biosynthetic background, and pharmacokinetic behavior. Understanding these additional aspects is important, as variations in plant origin, biosynthetic regulation, and metabolic fate contribute directly to the biological actions and therapeutic potential of the compound.

To address this gap, the present work provides a systematic review of Atr following PRISMA recommendations. The review examines its natural sources and chemical occurrence, current knowledge surrounding its biosynthesis, available pharmacokinetic evidence, and the reported pharmacological mechanisms and biological effects across different experimental systems. By integrating these dimensions, this study aims to give a consolidated and multidimensional view of Atr, clarify the state of current knowledge, and highlight research needs that may guide future pharmacological and biotechnological exploration.

## 2 Research Methodology

This systematic review adheres to the Preferred Reporting Items for Systematic Reviews and Meta‐Analyses (PRISMA) blueprints, ensuring a rigorous and transparent methodology. The general purpose of the work is to analyze pharmacological properties, natural sources, and biosynthesis of Atractylone through synthesizing results of peer‐reviewed articles. In December 2024, a complex literature search was conducted on four major databases (PubMed, Scopus, Web of Science, and Google Scholar) with an eye on the studies released by the time of that search. The search formula, using the right keywords, was (Atractylone) OR (Atractylon) significant of natural source, (biosynthesis) OR (biosynthetic pathway) and (biological activity). The keywords such as anticancer activity “OR,” “cancer,” “anti‐inflammatory,” “antioxidant” OR “oxidative stress,” “antiviral” OR “viral infection, pharmacokinetic, neuroprotective effect” OR “neurodegenerative,” and “gastrointestinal system protection” were also used to cover individual biological activities. Plaintext (AND/OR) flags were inserted to enhance sensitivity of search. No language or publication date restrictions were applied to maximize relevant data retrieval. A total of 427 articles were identified across all databases.

Study eligibility was determined using the PICO framework, with inclusion criteria focusing on original research articles that explored Atr biological activities, natural plant sources, and biosynthetic pathways. Studies utilizing in vitro, in vivo, or in silico approaches to evaluate its pharmacological properties were considered for inclusion.

Exclusion criteria were applied to studies that focused on unrelated compounds, lacked specific data on Atr's biological activities or biosynthesis, or were conference abstracts, editorials, or non‐peer‐reviewed literature. Duplicate studies identified across databases were also removed.

The selection process comprised an initial screening of titles and abstracts, followed by a comprehensive full‐text assessment to determine study eligibility. Relevant data were extracted from the included studies, focusing on study characteristics (author, year, country, type of study), biological activities (e.g., anticancer, anti‐inflammatory, antioxidant, antiviral, neuroprotective, gastrointestinal protection), natural sources of Atr, and biosynthetic pathway details (enzymes, precursors, and metabolic steps).

For data visualization, a word map of frequently occurring terms was generated using VOSviewer software (version 1.6.20) to identify key research trends. Chemical structures of Atr were generated using ChemDraw Pro 17.0, with their IUPAC nomenclature verified via the PubChem database. High‐quality graphical representations were created using BioRender Premium.

Due to the heterogeneity of study designs and reported outcomes, a narrative synthesis was performed to summarize the findings. The extracted data were systematically categorized based on biological activity type and biosynthetic mechanisms, facilitating a structured analysis of Atr's pharmacological potential and biosynthetic pathways.

## 3 Results and Discussion

**FIGURE 1:**
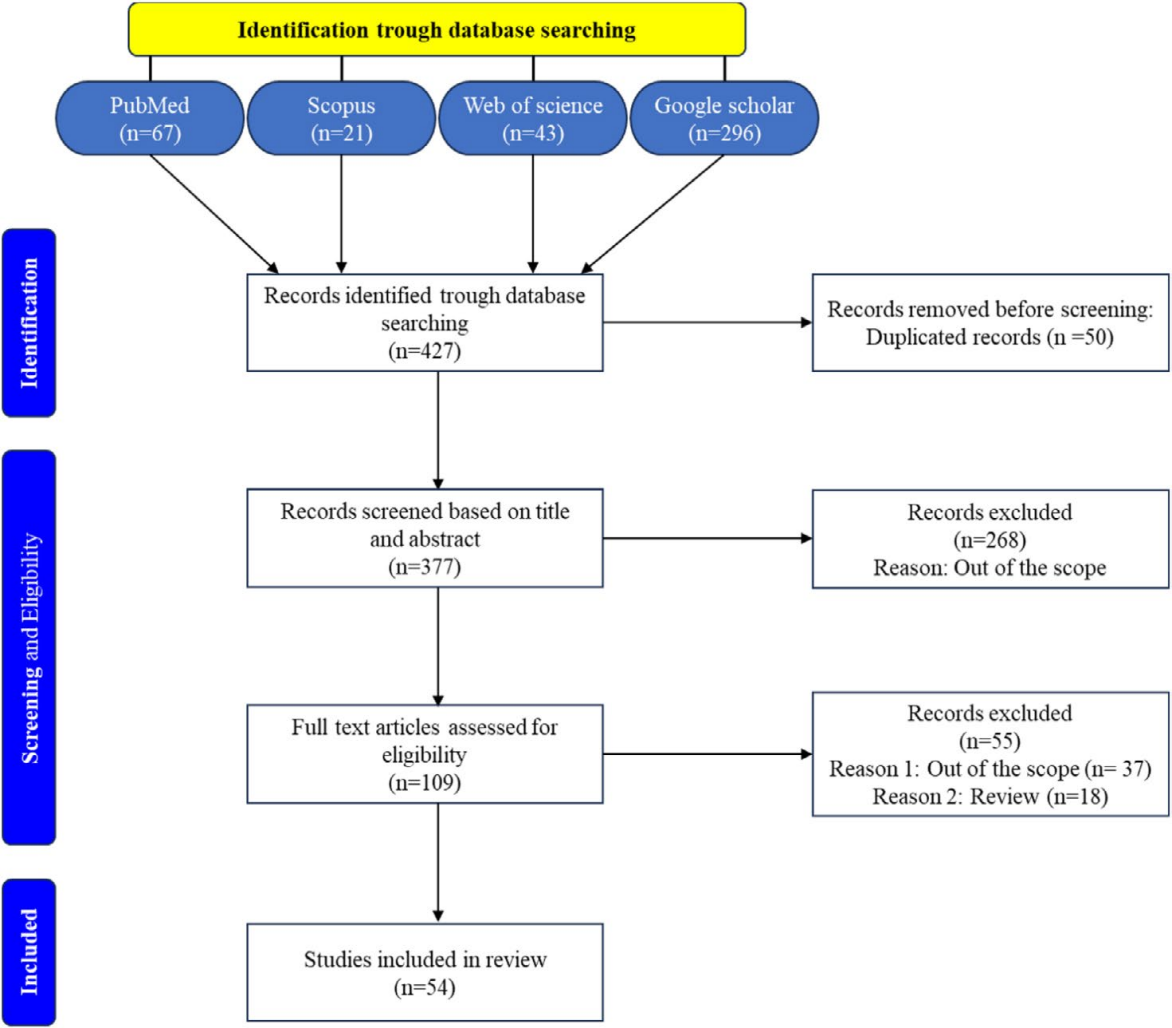
Overview of the study selection process, structured according to PRISMA guidelines.

A comprehensive literature search was conducted to characterize and valorize Atr, identifying 427 papers that met our inclusion criteria. To select the most relevant studies, we followed the PRISMA strategy, a systematic and transparent process involving the screening of titles and abstracts, eligibility assessment, and exclusion of papers that did not meet our criteria. As shown in Figure 1, after removing duplicate entries, we initially excluded 268 studies based on their titles, abstracts, and conclusions. A full‐text review was then conducted, leading to the exclusion of additional papers that were out of scope, lacked sufficient details, or did not meet the required rigor. Ultimately, 54 studies were included in this systematic review, providing a comprehensive analysis of Atr, including its natural sources, biosynthetic pathways, and biological activities.

**FIGURE 2:**
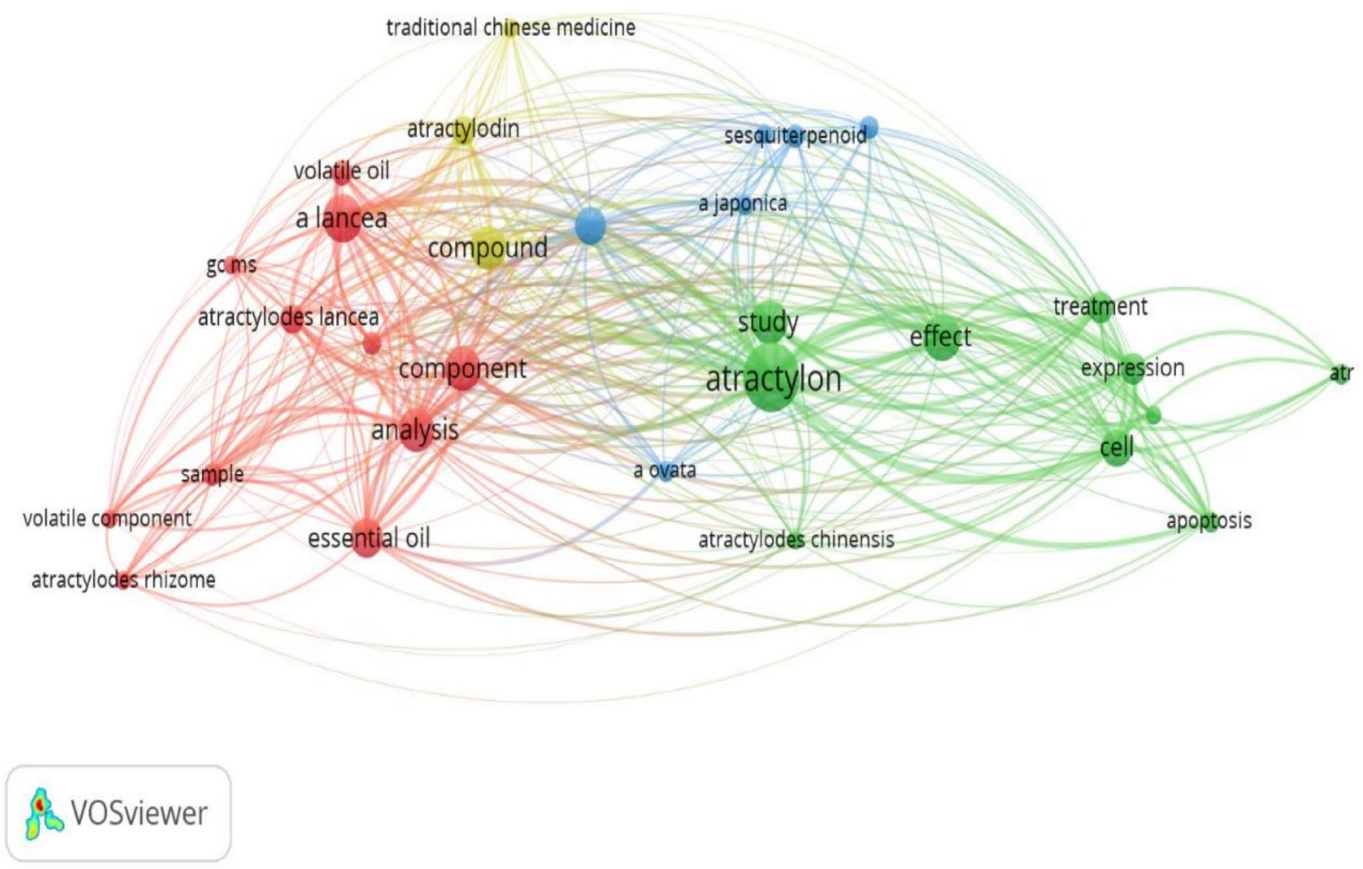
Term clustering map generated from the initial reference set of the systematic review before screening (423 references). Each color (red, green, blue, and yellow) represents a distinct cluster of terms. The size of each term node reflects its occurrence frequency, while the connecting lines represent the 100 most significant co‐occurrence relationships between terms.

As illustrated in Figure 2, the 30 terms in the word map are grouped into four clusters. The red cluster, consisting of 11 terms and located in the lower right of the figure, includes terms related to the natural sources of Atr, such as “study,” “plant,” “region,” “sample,” and “essential oil.” Other key terms in this cluster include “analysis” and “species.” The green cluster, which contains 10 terms and is positioned in the lower left, features key terms such as “Atractylon,” “effect,” “medicinal use,” and “pharmaceutical effects,” along with associated terms like “study,” “effect,” and “treatment.” The yellow cluster, composed of three terms and situated in the upper right, is primarily represented by “compounds,” “atractylodin,” and “traditional Chinese medicine.” The blue cluster, consisting of six terms and mainly located in the center, is characterized by “sesquiterpenoid,” “ *A. japonica* ,” and “ *A. ovata* .” Notably, “Atractylon” serves as a central linking term, connecting the red, green, yellow, and blue clusters, thereby bridging all four clusters.

### 3.1 Natural Sources of Atractylone

**FIGURE 3:**
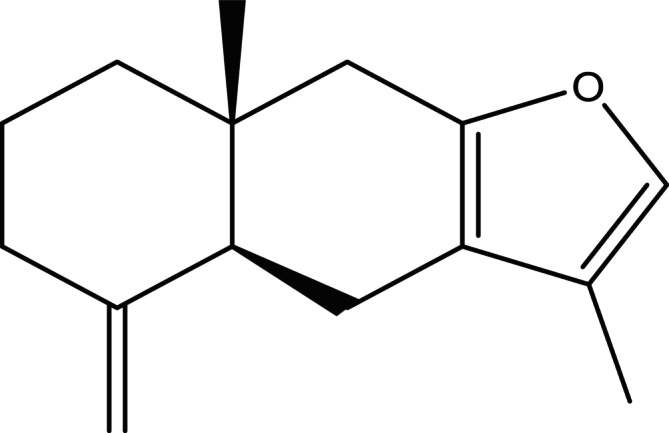
Chemical structure of Atractylone.

Atr is a volatile compound that comprises oxygenated sesquiterpenoids, which possess the molecular formula C_15_H_20_O (Zhang, Wang, et al. 2023). Structurally, it is characterized by a tricyclic framework consisting of two six‐membered carbon rings (cyclohexane systems) and one five‐membered oxygen‐containing ring (furan system), as illustrated in Figure 3 The presence of Atr may vary between the parts of plants and differ in different genera. In certain species it is located primarily in above ground parts and in others in underground structures (e.g., roots or rhizomes). The taxonomic distribution of Atr is summarized in Table 1 with concentration in Asteraceae family with preponderance in rhizomes. This family is majorly concentrated in areas such as China, Korea, Japan, India and Thailand. In the genus *Atractylodes*, contents of Atr are quite disparate, with a range of 0.30–338.5 mg/g among species (e.g., *A. japonica* , *A. lancea* , *A. ovata* , *A. koreana* , *A. chinensis* and *A. macrocephala* ). There are geographical factors affecting the occurrence and biomass of Atr in these plants (Chen et al. 2016; Li et al. 2013; Liu et al. 2016; Wang et al. 2002; Wang et al. 2023; Zhang, Wang, et al. 2023).

**TABLE 1:** The taxonomic distribution of Atractylone.

Species	Family	Part	References
*Atractylodes japonica*	Asteraceae	Rhizome	Chen et al. (2016)
*Atractylodes lancea*	Asteraceae	Rhizome	Zhang, Wang, et al. (2023)
*Atractylodes ovata*	Asteraceae	Rhizome	Wang et al. (2002)
*Atractylodes koreana*	Asteraceae	Rhizome	Liu et al. (2016)
*Atractylodes chinensis*	Asteraceae	Rhizome	Wang et al. (2023)
*Atractylodes macrocephala*	Asteraceae	Rhizome	Li et al. (2013)
*Siparuna guianensis*	Siparunaceae	Leaves	de Santana Oliveira et al. (2020)
*Siparuna thecaphora*	Siparunaceae	Fruit	Pino et al. (2009)
*Siparuna muricata*	Siparunaceae	Aerial parts	Burneo et al. (2021)
*Nectandra salicina*	Lauraceae	Leaves and branches	Ciccio et al. (2009)
*Ocotea lancifolia*	Lauraceae	Fruit (Seasonal variation)	da Thomas Silva et al. (2018)
*Cimicifuga simplex*	Ranunculaceae	Rhizome	Miyazawa and Kawata (2006)
*Callicarpa candicans*	Lamiaceae	Leaf and stem bark	Hung et al. (2020)
*Eugenia uniflora*	Myrtaceae	Leaves	Lago et al. (2011)
*Pinellia ternate*	Araceae	Rhizome	Iwasa et al. (2014)

Atr is also found in the aerial parts (e.g., leaves, branches and fruits) of the Siparunaceae family, that occurs in the regions of Nicaragua to Paraguay and within Brazil. It is found in the range of 11.51%–37.84% in the plants depending on the species and geographical distribution (Burneo et al. 2021; Pino et al. 2009; de Santana Oliveira et al. 2020). Equally, Nectandra, a genus in the family Lauraceae with a diurnal range of Florida to Argentina most of the species found in South America, has Atr in the leaves and branches of *N. salicina* as 14.6% of the total volatile compounds (Ciccio et al. 2009). *Ocotea lancifolia*, another species of Lauraceae, is the source of Atr fruits. Research shows that seasonal maturation influences the presence of Atr, the compound is expected in certain seasons and not in others. This fact indicates that Atr is integrated into the biosynthetic pathways of other derivative compounds in the chemical profile of the fruit (da Thomas Silva et al. 2018). More so, Atr was also detected in the chemical profiles of other species like *C. simplex* , *E. uniflora* , *C. candicans* , and *P. ternate* , the concentrations differed depending on the plant part and species (Hung et al. 2020; Iwasa et al. 2014; Lago et al. 2011; Miyazawa and Kawata 2006). Being a sesquiterpenoid of the terpene family, Atr is synthesized by complicated biosynthesis pathways. It has been found to possess a broad spectrum of biological activities that appealed to scientific attention.

### 3.2 Biosynthesis Pathway of Atractylone

Atr is a sesquiterpenoid derived from the universal C5 isoprenoid units isopentenyl diphosphate (IPP) and dimethylallyl diphosphate (DMAPP). These precursors are supplied primarily by the cytosolic mevalonate (MVA) pathway, with a subsidiary contribution from the plastidial methylerythritol phosphate (MEP) pathway. Both routes converge to form farnesyl diphosphate (FPP), the direct substrate for sesquiterpene cyclization (Zhang, Bai, et al. 2023).

Recent metabolomic and transcriptomic investigations in *Atractylodes* species have clarified how these pathways support Atr biosynthesis. In *A. chinensis* , 19 sesquiterpenes, including Atr, have been identified in the rhizome, and 29 full‐length genes related to terpene biosynthesis were detected through high‐throughput transcriptome sequencing. These findings indicate that sesquiterpene formation in the rhizome relies predominantly on the MVA pathway, although MVA/MEP crosstalk may contribute additional precursor flux (Zhang et al. 2025).

Within the MVA pathway, acetyl‐CoA is converted through a series of enzymatic steps involving Acetyl‐CoA acetyltransferase (AACT), 3‐Hydroxy‐3‐methylglutaryl‐CoA synthase (HMGS), 3‐Hydroxy‐3‐methylglutaryl‐CoA reductase (HMGR), Mevalonate kinase (MVK), Phosphomevalonate kinase (PMK), Mevalonate‐5‐diphosphate decarboxylase (MDC), and Farnesyl pyrophosphate synthase (FPPS). Among these, HMGR consistently emerges as a major regulatory bottleneck. Its importance has been demonstrated in several plant systems, and transcriptomic surveys in *Atractylodes* similarly report high HMGR expression in tissues with active terpenoid metabolism (Ahmed et al. 2016; Ruan et al. 2021).

The MEP pathway also contributes to IPP/DMAPP formation, although its quantitative involvement in Atr production remains uncertain. Tissue‐specific expression analyses show that MEP‐related genes, such as 1‐deoxy‐D‐xylulose‐5‐phosphate reductoisomerase (DXPS), are enriched in stems and leaves (Ruan et al. 2021), but the extent to which this influences rhizome‐localized sesquiterpene biosynthesis is still unresolved.

A major unknown step in Atr biosynthesis is the final cyclization of FPP into the eudesmane‐type skeleton characteristic of this compound. Several terpene synthases (TPS/STS) have been detected in *Atractylodes*, including three full‐length sesquiterpene synthase (STS) genes recently identified in *A. chinensis* , but none have been biochemically validated as the enzyme responsible for Atr formation (Zhang et al. 2025). These candidates may synthesize Atr, yet functional confirmation is still lacking and represents a central knowledge gap.

Environmental and genetic factors are known to influence the overall sesquiterpene profile in *Atractylodes*, although their direct relevance to Atr remains uncertain. Endophyte interactions, jasmonate signaling, and ecological variables have been associated with altered sesquiterpene production (Tsusaka et al. 2019; Wu et al. 2021; Yuan et al. 2019), largely by modulating precursor supply or TPS gene expression (Sun, Shi, et al. 2022). While these findings illustrate the regulatory flexibility of sesquiterpene metabolism, targeted studies are still needed to determine how they specifically affect Atr levels.

Overall, current evidence suggests that Atr biosynthesis proceeds mainly through the MVA pathway, driven by HMGR and FPPS activity, with subsequent cyclization mediated by an as yet unidentified sesquiterpene synthase. The discovery of candidate STS genes in *Atractylodes* provides a promising foundation for future functional work; however, until enzyme‐level validation is completed, the final steps leading to Atr remain largely inferred rather than experimentally confirmed.

### 3.3 Biological Activities of Atractylone

Atr is a naturally occurring volatile compound found in the chemical composition of various plant species (Table 1). However, data on its biological activity are insufficient, as it has not been extensively studied. In this systematic review, we aim to selectively examine and discuss the research conducted on this compound, as summarized in Table 2.

**TABLE 2:** Biological activities of Atractylone.

Biological activity	Method	Key results	References
Antioxidant activity	In vitro: DPPH	I% = 88%	Hwang et al. (1996)
	In vitro: RAW246.7 cells	↑ SOD ↑ GSH	Li et al. (2023)
Antiviral activity	In vitro: Influenza A/PR/8/34 virus (IAV)‐induced pulmonary Madin‐Darby canine kidney (MDCK) In vivo: IAV‐infected Male ICR mice	⊖ Attenuated IAV ↓ IL‐6 ↓ IL‐1β ↓ TNF‐α ↑ IFN‐β ↑ TRAF6 ↑ MyD88 ↑ TLR7 ↓ (NF‐κB) p65 ↑ IFN‐β mRNA	Cheng et al. (2016)
Anti‐inflammatory activity	In vitro: LPS‐stimulated RAW 264.7 cells In vivo: Male ICR mice	[I] of Cox‐2 = 25 μg/mL [I] of PGE_2_ = 25 μg/mL [I] of iNOS = 12.5 μg/mL ↓ Acetic acid‐induced	Chen et al. (2016)
	In vitro: BV2 cell line In vivo: LPS‐induced sepsis mouse model	↑ SIRT1 ↓ TNF‐α ↓ IL‐1β ↓ IL‐6 ↓ NF‐κB ↑ M2	Tian et al. (2019)
	In vitro: LPS‐induced RAW 264.7 cells	↓ Pro‐inflammatory cytokines	Li et al. (2023)
	In vitro: Inhibition of (*5‐LOX*) and (*COX‐1*)	IC_50_ (*5‐LOX*) = 25.1 μM IC_50_ (*COX‐1*) = 200 μM	Resch et al. (1998)
	In silico: Molecular docking of Atr with muscarinic acetylcholine receptor M3 (CHRM3)	Strong binding affinity with isoleucine (ILE)‐222 Tyrosine (TYR)‐148	Yang et al. (2020)
	In vivo: TPA‐induced inflammation in mice	ID_50_ = 0.9 mg/ear	Yu et al. (1994)
	In vitro: PMACI‐treated HMC‐1 cells	↑ TNF‐α ↑ IL‐6 ↑ IL‐8 ↑ IL‐1 ↑ TSLP	Kim et al. (2016)
	In vitro: LPS‐stimulated ANA‐1 macrophages	↓ NO	Gu et al. (2019)
	In vitro: HMC‐1 cell line In vivo: Male Sprague Dawley rats (7 weeks old) and ICR mice (4 weeks old)	↓ Calcium influx ↓ p56lck tyrosine kinase ↓ IL‐13, IL‐4, IL‐5 and IL‐6 ↓ Serum levels of histamine	Han et al. (2016)
	In silico: Docking molecular Networking pharmacology	TNF = −7.9 kcal/mol IL‐6 = −6 kcal/mol IFN‐γ = −6.1 kcal/mol IL‐1β = −5.7 kcal/mol IL‐10 = −7.3 kcal/mol IL‐4 = −6.1 kcal/mol TLR9 = −6.8 kcal/mol	Wu and Hu (2020)
Anticancer activity	In vitro: GBM cell line	↑ G1 phase ↓ G2/M phases ↓ S phase ↑ Caspase‐3 ↑ mRNA levels of *SIRT3*	Sun, Shi, et al. (2022); Sun, Luo, et al. (2022)
	In vitro: HepG2 SMCC7721 MHCC97H cell line In vivo: Mice‐xenograft models	IC_50_ = 26.18 μM IC_50_ = 22.32 μM IC_50_ = 34.14 μM ↑ Apoptosis ↓ *Bcl‐2* ↑ *Bax* ↑ caspase‐3 ↑ E‐cadherin ↓ α‐SMA ↓ Vimentin ↓ N‐cadherin ↓ MMP‐2/9	Cheng et al. (2019)
	In vitro: HepG2 cell line In vivo: BALB/c nude mice	↑ TMPo‐aS1 ↓ Ccdc183‐aS1	Cheng et al. (2022)
	In vitro: Intestinal cancer HT29 cell line	↓ INF‐γ ↓ TNF‐α ↓ MMP‐9 ↓ Bcl‐2 ↓ PI3K ↓ AKT ↓ mTOR ↑ Apoptosis	Mao et al. (2022)
	In vivo: DMBA‐ and TPA‐induced skin tumor formation in mice	↓ 96% of tumors per mouse	Yu et al. (1994)
	In vitro: HepG2 MCG803 HCT‐116 cancer cell line	IC_50_ = 90 μg/mL IC_50_ = 70 μg/mL IC_50_ = 25 μg/mL	Gu et al. (2019)
	In vitro: HepG2 cell line	IC_50_ = 25 μmol/mL ↑ Caspase‐3 ↓ Bcl‐2 ↑ Bax	杨雪丽 et al. (2021)
Neuroprotective activity	In vitro: 293T‐Tango‐PB cells In vivo: C57BL/6 mice at the age of 12 weeks	↓ cAMP ↑ eGFP ↑ Phosphorylated CREB/BDN ↓ T‐climbing time ↓ T‐turning time	Li, Wang, et al. (2022); Li, Du, et al. (2022)
	In vivo: Male C57BL/6 mice At the age of 4–6 weeks	↓ Neuronal apoptosis	Tian et al. (2019)
	In silico: Molecular docking (GluN1, GluN2B, and GluA2) glutamate receptor subunits In vitro: PC12 cells In vivo: Male ICR outbred mice at the age of 5 weeks	GluN1 = −8.7 kcal/mol GluN2B = −8.8 kcal/mol GluA2 = −6.1 kcal/mol ↓ LDH ↓ TNF‐α ↓ NF‐κB ↑ TST ↑ EPM ↑ SIRT1 ↓ IL‐6	Tran et al. (2023)
Gastrointestinal protective effects	In vitro: IEC‐6 cell line	↑ [Ca^2+^]cyt ↑ STIM1 ↑ TRPC1 ↑ PLC‐γ1 ↑ RhoA	Ren et al. (2021)
	In vivo: Sprague Dawley (SD, 6–8 weeks) rats	↓ TNF‐α ↓ NO ↑ SOD ↑ IL‐10 ↓ MDA ↑ Microbiota diversity	Li, Wang, et al. (2022), Li, Du, et al. (2022)
Pharmacokinetic	The pharmacokinetic profile of Atr was characterized through noncompartmental analysis of plasma concentration‐time data using DAS 2.1 pharmacokinetic software.	*C*_max_ = 257.1 ng/mL *t*_max_ = 1.83 h *t*_1/2_ = 7.64 h MRT_0−∞_ = 9.79 h AUC_0−∞_ = 1533.7 ng h/mL	Yan et al. (2015)
	In vivo: The pharmacokinetic profiles of the volatile constituents were evaluated in Sprague–Dawley rats (six males and six females) using noncompartmental analysis performed with Phoenix WinNonlin 6.1 software	*C*_max_ = 721.33 ng/mL AUC_0−t_ = 2230.4 ng h/mL *t*_max_ = 2 h *t*_1/2_ = 4 h	Zan et al. (2022)

Abbreviations: [I] = concentration of inhibition, ⊖ = blocked, ↑ = increased, ↓ = decreased, 5‐LOX = 5‐lipoxygenase, AUC = area under the concentration‐time curve., cAMP = cyclic adenosine monophosphate, *C*_max_ = peak plasma concentration, COX‐1/COX‐2 = cyclooxygenases, CREB/BDNF = cAMP response element‐binding protein/brain‐derived neurotrophic factor, GSH = glutathione, I% = inhibition percentage, IC_50_ = half‐maximal inhibitory concentration, ID_50_ = median inhibitory dose, IFN‐β/IFN‐γ = interferons, IL‐1β/IL‐6/IL‐8/IL‐10/IL‐13 = interleukins, iNOS = inducible nitric oxide synthase, LDH = lactate dehydrogenase, MDA = malondialdehyde, MRT = mean residence time, MyD88 = myeloid differentiation primary response 88, NF‐κB = nuclear factor kappa‐B, PGE2 = prostaglandin E2, PLC‐γ1 = phospholipase C‐gamma‐1, SOD = superoxide dismutase, STIM1 = stromal interaction molecule 1, *t*½ = elimination half‐life, TLR7/TLR9 = Toll‐like receptors, *t*_max_ = time to reach *C*_max_, TNF‐α = tumor necrosis factor alpha, TRAF6 = TNF receptor‐associated factor‐6, TRPC1 = transient receptor potential channel 1.

#### 3.3.1 Antiviral Activity

A recent study on the antiviral properties of the Atr, a bioactive compound of *Atractylodis rhizoma*, employed in vitro and in vivo experimental approaches. Data revealed that treatment with Atr (10–40 mg/kg during 5 days) against LD50 dose of the influenza A virus (IAV) reduced pulmonary insult. This protective influence was associated with the lowering of serum tumor necrosis factor‐alpha (TNF‐alpha), IL‐1 beta, and IL‐6 combined with production of interferon‐beta (IFN‐beta). In vivo analysis also indicated that treatment with Atr increased the level of expression of *Toll‐like receptor 7* (*TLR7*), *tumor necrosis factor receptor‐associated factor 6* (*TRAF6*), *MyD88* and *IFN‐β* mRNA but had a down‐regulatory effect on nuclear factor‐κB (NF‐κB) p65 protein in the lung tissues of mice infected with IAV. These findings suggest that Atr is a strong antiviral agent via its immune signaling pathway modulation properties, which brings forth its use as an antiviral therapeutic agent (Cheng et al. 2016). However, the opposite account was given by Gu et al. (2019) who discovered that Atr was mostly ineffective with the influenza H3N2 strain. The authors provided a reasonable explanation for this discrepancy, pointing to the low concentration used as the primary factor (Gu et al. 2019). This finding critically underlines that Atr efficacy is probably very dose‐dependent, and strong effects observed on one strain might need a higher or lower dosing regimen against other strains.

Taken together, these results indicate that Atr is an antiviral that acts mainly by mediating via immunomodulation as opposed to having direct virucidal action. This creates the image of Atr as a host‐directed therapeutic, making the immune response fine‐tuning to eliminate the infection successfully.

#### 3.3.2 Antinociceptive Activity

Research into the antinociceptive properties of Atr reveals a consistent analgesic effect across different plant sources and experimental models, though with varying apparent potency. The foundational study by Amorim et al. (2009), first identified Atr as a key bioactive furanosequiterpene in *E. uniflora* L., where it, along with 3‐furanoeudesmen, was responsible for the potent antinociceptive effect of the pentane fraction. Notably, these isolated compounds achieved the same efficacy (70% inhibition) as the whole fraction but at half the dose (100 mg/kg), suggesting that Atr is a primary driver of the observed activity (Amorim et al. 2009). This finding is corroborated and refined by a later study on *A. japonica* by Chen et al. (2016); their work not only confirmed Atr's analgesic effect but also demonstrated its action in a dose‐dependent manner at significantly lower doses (10–40 mg/kg). Furthermore, Chen and their team expanded the mechanistic understanding by employing both a chemical (acetic acid) and a thermal (hot plate) model of pain. The efficacy in the hot plate test, in particular, suggests a potential central nervous system mechanism, moving beyond a purely peripheral anti‐inflammatory action (Chen et al. 2016).

When synthesized, the evidence from these two studies paints a compelling picture. The fact that Atr is effective in different plant extracts points to its fundamental role as an analgesic compound. However, the considerable difference in effective doses between the studies (100 and 10–40 mg/kg) warrants attention. This discrepancy could be attributed to differences in bioavailability from the plant matrix, the specific pain models used, or the route of administration. Collectively, the data strongly position Atr as a promising analgesic agent with activity in multiple pain pathways, though future studies are needed to precisely elucidate its primary mechanism of action and clarify its dose–response relationship.

#### 3.3.3 Antioxidant Activity

Redox imbalance has been considered one of the major factors of many chronic diseases that are associated with the disturbance of redox homeostasis. This disequilibrium is typified by an increase in the level of reactive oxygen species (ROS) and a reduction of antioxidant substances like catalase and superoxide dismutase (SOD) that also promotes the spread of the disease. A study was conducted to determine the protective effect of Atr on the damage of DNA and on hepatotoxicity within the hepatocytes of rats that were induced using tert‐butyl hydroperoxide. Its outcome shows great antioxidant properties by showing that Atr decimates nearly 88% of DPPH radicals at 1.0 mg/mL. Moreover, at the doses of 0.01, 0.1 and 1.0 mg/mL Atr inhibited malondialdehyde (MDA) and lactate dehydrogenase (LDH) leakage, showing the defensive effect of Atr against oxidative damage to hepatocytes (Hwang et al. 1996). This profile is reinforced and expanded by more recent research in an inflammatory context. Li et al. (2023) demonstrated that in LPS‐induced RAW264.7 macrophages, Atr significantly reduced ROS generation and, more importantly, actively boosted the cellular antioxidant defense system by inducing key enzymes like SOD and glutathione (GSH) (Li et al. 2023).

When synthesized, the evidence from these two studies paints a coherent and compelling picture of Atr's multifaceted antioxidant mechanism. The research by Hwang et al. primarily reveals Atr's role as a direct scavenger and membrane protector, while the work of Li et al. suggests it can also upregulate the body's own endogenous antioxidant machinery. However, a critical gap remains. Both studies are preclinical and in vitro; the promising dose‐dependent effects observed in cells must be interpreted with caution until confirmed in vivo, where factors like bioavailability and metabolism come into play. Collectively, the data robustly position Atr as a promising antioxidant agent, but its therapeutic potential hinges on future validation in whole‐organism models.

#### 3.3.4 Anti‐Inflammatory Activity

Numerous studies have explored the anti‐inflammatory activity of Atr using in vitro, in vivo, and in silico approaches. In one study, Atr significantly inhibited key inflammatory mediators. In LPS‐stimulated RAW 264.7 macrophages, Atr reduced the production of nitric oxide (NO) and prostaglandin E2 (PGE2), as well as the expression of inducible nitric oxide synthase (iNOS) and cyclooxygenase‐2 (COX‐2). In vivo, administration of Atr (40 mg/kg) reduced acetic acid‐induced writhing, decreased carrageenan‐induced paw oedema, and prolonged nociceptive latency in the hot plate test. These results highlight the strong anti‐inflammatory and antinociceptive potential of *A. japonica* , with Atr identified as a key active component (Chen et al. 2016). This foundational finding of broad anti‐inflammatory activity is complemented by research delving into specific molecular pathways. Another study examined the neuroprotective effects of Atr in sepsis‐associated encephalopathy and cognitive impairment. Cognitive function was assessed in mice with LPS‐induced sepsis using the Morris water maze and open field test, while BV2 microglial cells were used to explore cellular mechanisms. Atr exerted anti‐inflammatory effects through the SIRT1/NF‐κB signaling pathway. LPS reduced SIRT1 expression and activated NF‐κB; treatment with Atr reversed these effects by upregulating SIRT1, which suppressed NF‐κB expression. Deactivation of SIRT1 blocked this suppression, confirming that Atr regulates NF‐κB via SIRT1. Flow cytometry revealed that Atr prevented LPS‐induced microglial apoptosis, and ELISA showed a reduction in TNF‐α, IL‐6, and IL‐1β production, effects that were reversed when SIRT1 was inhibited. Furthermore, Atr promoted the transition from pro‐inflammatory M1 microglia to anti‐inflammatory M2 microglia via SIRT1 activation (Tian et al. 2019). Beyond the central nervous system, the anti‐inflammatory effects of Atr show consistency across different cell types and models, reinforcing its broad applicability. Atr also exhibits dose‐dependent anti‐inflammatory effects. In LPS‐induced RAW 264.7 cells, it suppressed the secretion of IL‐6 and TNF‐α (Li et al. 2023). It acts as a selective inhibitor of 5‐lipoxygenase (5‐LOX) with an IC_50_ of 25.1 μM, showing minimal activity against COX‐1 (> 200 μM) (Resch et al. 1998). The relevance of these isolated findings is strengthened by their confirmation in more complex, clinically relevant systems. Analysis of TCM formulas, such as the Ermiao San (ESSF) and Sanhan Huashi (SHHS) series, revealed that Atr contributes significantly to their anti‐inflammatory activity by reducing cytokines such as CCL‐2, TNF‐α, IL‐6, CCL‐3, and CXCL‐10 in SARS‐CoV‐2‐infected Vero‐E6 cells (Liu et al. 2024; Tian et al. 2024). Molecular docking studies have demonstrated that Atr binds to the muscarinic acetylcholine receptor M3 (CHRM3), interacting with ILE‐222 and TYR‐148, which could reduce acetylcholine‐mediated inflammatory signaling (Yang et al. 2020). This multi‐target potential is further illustrated by Atr's efficacy in specific disease models, which also reveals its ability to modulate complex fibrotic and allergic processes. In a mouse model of ovalbumin‐induced pulmonary fibrosis, Atr modulated fibrosis‐related proteins by downregulating circRNA‐0000981 and TGFBR2 while upregulating miR‐211‐5p, thereby suppressing TGF‐β1‐induced epithelial‐mesenchymal transition (EMT) and fibrosis (Zeng et al. 2021). Additional studies in TPA‐induced murine inflammation showed that Atr inhibited ear edema with an ID_50_ of 0.9 mg/ear, with anti‐inflammatory efficacy comparable to cepharanthine but less potent than hydrocortisone or indomethacin (Yu et al. 1994). A critical aspect of Atr's activity is its targeted action on mast cells and its context‐dependent nature, which distinguishes it from a general immunosuppressant. Atr suppressed inflammatory cytokines production induced by PMACI, which emphasizes its potential in the control of allergic inflammatory reactions. Atr demonstrated dose‐dependent inhibition against pro‐inflammatory cytokine gene expression in PMACI‐stimulated HMC‐1 cells, which include, but are not limited to, thymic stromal lymphopoietin (TSLP), interleukin IL‐6, IL‐1, IL‐8, and tumor necrosis factor‐alpha (TNF‐α). Also, quantitative real‐time PCR confirmed that Atr decreased mRNA expression of these cytokines induced by PMACI in a dose‐dependent manner. Nonetheless, Atr itself did not modulate the generation of inflammatory cytokines relative to media control, and DMSO did not modulate the production of cytokines in PMACI‐stimulated cells. These observations suggest that Atr stimulates its anti‐inflammatory effects following inflammatory signals, but not by the more general repression of basal cytokines (Kim et al. 2016). Similarly, Atr reduced NO production in LPS‐stimulated ANA‐1 macrophages without cytotoxicity (Gu et al. 2019). This potent anti‐allergic profile is consistently linked to the direct suppression of mast cell activation, a key driver in allergic diseases. Atr, a compound with bioactive properties of PyeongweeSan (KMP6), possesses strong anti‐allergic and anti‐inflammatory activities based on regulation of immune functions related to the mast cells actions. This experiment indicated that Atr can significantly prevent mast cell degranulation both in the in vitro and in vivo models. Atr inhibited intracellular calcium entry, release of tryptase and histamine, and p56lck tyrosine kinase activity a key regulator of mast cell activation in rat peritoneal mast cells (RPMCs) induced by compound 48/80 and human mast cells (HMC‐1) stimulated by PMA and A23187. Also, Atr notably ameliorated the IgE‐mediated passive cutaneous anaphylaxis (PCA) in mice by reducing the serum concentration of histamine, IgE, IL‐6, IL‐5, IL‐13, IL‐4, and vascular endothelial growth factor (VEGF). In addition, Atr reduced systemic anaphylaxis induced by compound 48/80 along with ear edema, which was supportive of its use in managing allergic inflammation. These results indicate that Atr has potential therapeutic applications as the allergy treatment since it also interferes with mast cell activation and controls the release of inflammatory mediators (Han et al. 2016). Network pharmacology approach study explored the system of active compounds of *A. macrocephalae* Rhizoma against pneumonia. Seven predicted targets associated with inflammation and immune responses were identified using various bioinformatics assays, which include IL‐10, IL‐6, TLR9, IL‐1B, TNF, IL‐4, and IFN‐silrgamma. Out of the active compounds, Atr was demonstrated to have actions on these targets endorsing this mitigation of immune responses by inhibiting activated inflammatory cytokines. Regulatory effects of Atr on pneumonia were closely connected with the interaction between inflammation and the immune response (Wu and Hu 2020).

Overall, these studies indicate that Atr exhibits potent anti‐inflammatory activity by targeting key signaling pathways, reducing pro‐inflammatory cytokines, preventing cell apoptosis, and modulating microglial polarization. These properties suggest its therapeutic potential in managing inflammation‐related diseases.

#### 3.3.5 Anticancer Activity

**FIGURE 4:**
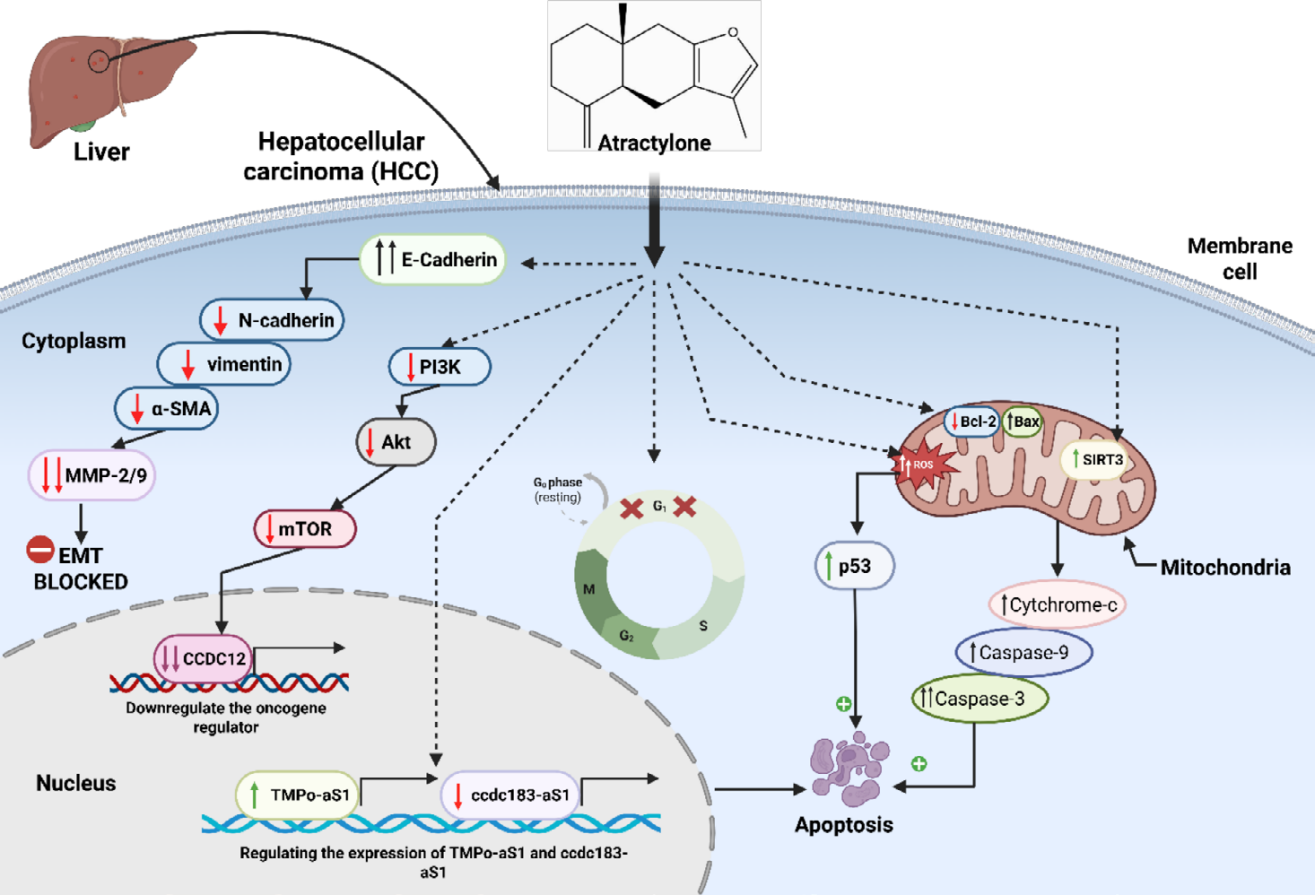
Anticancer mechanisms of Atr in hepatocellular carcinoma (HCC), highlighting its ability to inhibit proliferation, metastasis, and induce apoptosis. Atractylone blocks the EMT by increasing E‐cadherin and reducing MMP‐2/9, preventing cancer cell migration and invasion. It also downregulates the PI3K/AKT/mTOR pathway, leading to cell cycle arrest at the G1 phase, which halts cancer cell division. Additionally, Atr suppresses oncogene activity by downregulating CCDC12, reducing cancer progression. In the mitochondria, Atr induces oxidative stress (ROS), decreases Bcl‐2 (anti‐apoptotic), and increases Bax (pro‐apoptotic), leading to mitochondrial membrane permeabilization and activation of caspase‐3, a key apoptosis effector. These combined mechanisms result in cancer cell death, making Atr a promising therapeutic candidate for HCC treatment.

Atr has attracted considerable scientific interest due to its numerous biological activities, particularly its anticancer potential. Essential oils containing Atr exhibit potent anti‐proliferative effects, prompting researchers to investigate its role as a key bioactive compound. In glioblastoma multiforme (GBM) cells, Atr suppressed cell viability and proliferation, induced cell cycle arrest in the G1 phase, and promoted apoptosis. Flow cytometry revealed an increase in the proportion of C6 cells in the G1 phase, with a corresponding reduction in the G2/M and S phases. Pro‐apoptotic markers, including cleaved caspase‐3, were upregulated, and the migration of C6 and DBTRG cells was significantly inhibited. Atr also enhanced SIRT3 expression at the mRNA and protein levels, thereby contributing to the inhibition of tumorigenesis. PH3 staining confirmed a reduction in tumor proliferation after Atr treatment, collectively demonstrating its anticancer effects via SIRT3 activation in GBM cells (Sun, Luo, et al. 2022; Sun, Shi, et al. 2022). The anticancer potential of Atr is not limited to brain cancers, showing equally compelling activity against hepatic carcinomas through distinct mechanisms. Similarly, Atr shows promising activity against liver carcinoma. In vitro, it inhibited the proliferation of HepG2, SMCC7721, and MHCC97H cells in a concentration‐dependent manner, with IC_50_ values of 26.19, 22.32 and 34.14 μM, respectively. Flow cytometry indicated increased apoptosis, with downregulation of Bcl‐2 and upregulation of Bax and cleaved caspase‐3, confirming activation of the mitochondrial pathway (Figure 4). In xenograft models, Atr reduced tumor growth by decreasing Ki‐67 expression and modulating EMT markers: increasing E‐cadherin while decreasing α‐SMA, vimentin and N‐cadherin (Cheng et al. 2019). Building on this foundation, subsequent research has begun to pinpoint the specific genetic targets through which Atr exerts its effects in liver cancer. Indeed, the same research team conducted a deeper investigation to identify the key genes involved in the mechanism of action of Atr. Their findings revealed that Atr exerts its anticancer effects in hepatic carcinoma by regulating the expression of *TMPo‐aS1* and *ccdc183‐aS1*, two genes implicated in tumor progression. In vitro, Atr inhibited cell invasion and migration, while in vivo, it upregulated *TMPo‐aS1* and downregulated *ccdc183‐aS1*, suggesting their distinct roles in cancer suppression. These results highlight *TMPo‐aS1* and *ccdc183‐aS1* as potential molecular targets for liver cancer identification and therapeutic intervention, offering new therapeutic insights (Cheng et al. 2022). This theme of pathway‐specific inhibition is a consistent thread, as demonstrated in colorectal cancer models where Atr targets a well‐known oncogenic cascade. In addition, an independent study revealed that Atr regulates cell proliferation in a dose‐dependent manner. At a concentration of 15 mg/mL, a reduction in cell density was observed, while cell morphology remained largely intact. However, treatment with 30 mg/mL of Atr resulted in a significant decrease in cell number, a marked reduction in proliferative activity, and a marked increase in apoptosis. In addition, the proportions of pro‐inflammatory cytokines INF‐γ, TNF‐α, and MMP‐9 were significantly reduced in the 30 mg/mL treatment group. The expression of key survival and proliferative signaling markers, including *AKT*, *Bcl‐2*, *mTOR*, and *PI3K*, was significantly reduced in this group, while these markers were upregulated in the negative control group. Conversely, caspase‐3 expression was significantly elevated after treatment with 30 mg/mL of Atr, indicating increased apoptotic activity. These results suggest that Atr effectively suppresses the proliferation of intestinal cancer cells and induces apoptosis through a dose‐dependent suppression of the *PI3K/AKT/mTOR* signaling cascade. This mechanism suggests its potential use in the treatment of colorectal cancer and related diseases (Mao et al. 2022). Beyond therapeutic intervention, Atr also demonstrates significant potential in cancer prevention, as shown in classic carcinogenesis models. Atr also demonstrates strong chemopreventive effects in vivo. In a DMBA‐ and TPA‐induced skin tumor model, control mice developed tumors starting at week 6, with 100% incidence by week 20 and an average of 10.4 tumors per mouse. Treatment with 21 μmol Atr delayed tumor onset to week 8, with only 33% of mice developing tumors and an average of 0.4 tumors per mouse, representing a 67% reduction in incidence and 96% reduction in tumor count, highlighting its potential in skin cancer prevention (Yu et al. 1994). When comparing isolated Atr to its natural essential oil mixtures, a nuanced picture of its efficacy emerges, important for understanding its role in traditional medicine. In vitro cytotoxicity experiments showed that Atr and essential oils (EO) derived from crude *A. macrocephala* (CA) and bran‐treated *A. macrocephala* (BA) inhibited the proliferation of HepG2, MCG803, and HCT‐116 cancer cells. Atr exhibited the strongest antitumor activity, with lower IC_50_ values in HepG2 and HCT‐116 cells compared to EOs. Furthermore, EOs derived from CA were more effective than those derived from BA in inhibiting the proliferation of HepG2 cells. These results suggest that Atr is a potent anticancer agent, particularly against liver and colon cancer, and that EOs from CA have a stronger cytotoxic effect than those from BA (Gu et al. 2019). The reproducibility of Atr's pro‐apoptotic effects in liver cancer cells is a key strength, with multiple independent studies confirming the involvement of the mitochondrial pathway. Atr caused a significant, dose‐ and time‐responsive reduction in cellular survival, with an IC_50_ of 26.19 μmol/L in HepG2 cells after 72 h. It increased apoptosis rates, elevated ROS levels, and inhibited cell migration after 48 h. Additionally, Atr downregulated the anti‐apoptotic protein Bcl‐2 while upregulating Bax and cleaved caspase‐3, confirming apoptosis induction (杨雪丽 et al. 2021). Finally, the scope of Atr's activity extends to other hard‐to‐treat cancers, suggesting a broad‐spectrum potential that merits further exploration. Atr's anticancer effects extend to other carcinoma models as well. It demonstrated cytotoxicity against P‐388 cells, induced apoptosis after 15 μg/mL treatment, and is a major constituent of Alpinia (AL) extract, which shows anti‐cholangiocarcinoma activity through apoptosis, cell cycle arrest, and cytotoxicity. Atr, present at 2% in the extract, may contribute additively or synergistically to its overall anticancer effects. Given the heterogeneity of cholangiocarcinoma subtypes, Atr's therapeutic potential warrants further investigation (Martviset et al. 2018; Wang et al. 2002, 2007, 2011; Zhuang et al. 2022). Collectively, these studies consistently demonstrate that Atr exerts antitumor activity by activating apoptotic pathways, inhibiting proliferation, modulating gene expression, and suppressing metastatic potential in several types of cancer, highlighting its promising potential as a therapeutic agent.

#### 3.3.6 Neuroprotective Activity

Atr demonstrates significant neuroprotective activity, particularly in Parkinson's disease (PD), by modulating dopaminergic signaling and attenuating neurotoxicity. In vitro, Atr decreased cAMP production in a dose‐dependent manner (IC_50_ = 2.5 μM) and induced eGFP expression, similar to the DRD2 agonist cabergoline, suggesting that it may act as a novel DRD2 agonist. It further upregulated phosphorylated CREB and BDNF, two downstream DRD2 targets essential for neuronal survival and synaptic plasticity. In vivo, Atr alleviated motor dysfunction in an MPTP‐induced PD mouse model, significantly reducing T‐turning and T‐climbing times in the pole test, with effects comparable to pramipexole (PPX). Since MPTP metabolism generates MPP^+^, which selectively accumulates in dopaminergic neurons, Atr's protective activity is particularly relevant in preventing substantia nigra pars compacta (SNpc) degeneration and striatal dopamine depletion. Quantitative analysis revealed a significant preservation of tyrosine hydroxylase (TH)‐positive neurons in Atr‐treated mice, confirming dose‐dependent neuroprotection (Li, Wang, et al. 2022). This targeted action on dopaminergic neurons in PD is complemented by its broader role in combating systemic inflammation‐driven brain injury. Beyond PD, Atr also provides neuroprotection in sepsis‐associated encephalopathy (SAE). In experimental SAE models, Atr reduced neuronal apoptosis, alleviated cognitive impairments, and suppressed neuroinflammation, collectively mitigating sepsis‐induced brain dysfunction (Tian et al. 2019). Furthermore, Atr's neuroprotective profile extends beyond degenerative and inflammatory conditions to include complex mood disorders, revealing a multi‐receptor mechanism of action. Molecular docking studies revealed strong binding affinity of Atr for GluN1, GluN2B, and GluA2 glutamate receptor subunits, exceeding that of ketamine. This suggests that Atr can modulate glutamate receptor signaling, a key mechanism underlying both neuroprotection and rapid antidepressant effects. In vitro, essential oils containing Atr protected neural cells from corticosterone (CORT)‐induced cytotoxicity by reducing lactate dehydrogenase (LDH) leakage, thereby preserving neuronal integrity. In LPS‐challenged models, Atr reduced TNF‐alpha and IL‐6 levels which are indicative of anti‐inflammatory trends. Its antidepressant and anxiolytic properties are also evidenced in vivo. Essential oils with Atr displayed antidepressant‐like effect (shorter immobilization time in the TST) and anxiolytic‐like effect (increased entries in the open‐arm of the EPM). Mechanistically, Atr phosphorylated SIRT1 and inhibited the NF‐κB pathway, resulting in decreased inflammation, increase in the survival of neurons, and mood improvement (Tran et al. 2023). Together these findings point to Atr as a potentially powerful therapeutic agent of neurodegenerative diseases and mood disorders. By acting through various mechanisms (dopaminergic, glutamatergic, and inflammatory pathways), Atr would not only provide neuroprotection against neurodegenerative diseases such as Parkinson, severe anxiety disorders, and stress‐related brain damage, but also be an antidepressant and anxiolytic agent. The importance of its neuroprotective effects can be deeply connected with its overall anti‐inflammatory effects pointing to its benefits as an effective treatment of not only cognitive impairment, but also neuroinflammation in multiple neurological disorders.

#### 3.3.7 Gastrointestinal Protective Activity

The gastrointestinal (GI) tract functions as a critical barrier between the external environment and internal systems, with its integrity essential for maintaining homeostasis. Natural bioactive compounds with protective effects on GI disorders have received increasing interest, and among them, Atr has been found to have a favorable potential. It was revealed that Atr enhances intestinal epithelial healing by activating the Ca^2+^ signaling pathway, with Atr raising intracellular calcium concentration, increasing expression of STIM1 and TRPC1, PLC‐gamma 1, and Rho A, and promoting migration and proliferation of IEC‐6 cells through cell‐cycle progression (Ren et al. 2021). This detailed mechanistic insight into cellular repair is powerfully complemented by in vivo evidence demonstrating Atr's systemic protective effects against common GI insults. Complementary findings by Li, Wang, et al. (2022); Li, Du, et al. (2022) revealed that Atr protects against *H. pylori* and ethanol‐induced mucosal injury through its antioxidant, anti‐inflammatory, and microbiota‐modulating actions. Atr reduced ulceration, oxidative stress, neutrophil infiltration, TNF‐α, and NO levels, while restoring IL‐10, SOD, and gut microbial diversity—particularly enriching Bacteroides, Ruminococcus, and Parabacteroides. When viewed together, these studies present a compelling, multifaceted picture of Atr's gastroprotective role. Collectively, these studies highlight Atr as a multifunctional agent with therapeutic potential for gastrointestinal protection and ulcer management (Li, Du, et al. 2022).

The research by Ren et al. elucidates the fundamental pro‐healing mechanism at the cellular level, while Li et al. confirms the protective outcome at the whole‐tissue level, showcasing how Atr acts not only to repair damage but also to prevent it through antioxidant, anti‐inflammatory, and even prebiotic activities.

#### 3.3.8 Pharmacokinetics of Atractylone

Pharmacokinetic studies demonstrate that Atr is effectively absorbed after oral administration. In rats, Atr reached a maximum plasma concentration (*C*_max_) of 257.1 ng/mL in 1.83 h (*t*_max_) and had a half‐life (*t*_1/2_) of 7.64 h with a mean residence time (MRT_0−∞_) of 9.79 h, resulting in systemic exposure (AUC_0−∞_) of 1533.7 ng·h/mL (Yan et al. 2015). This favorable standalone profile is intriguingly modified when Atr is administered within a traditional medicinal formula, highlighting the impact of herbal synergy. Another study investigated the pharmacokinetic mechanism of Atr in the popular medicinal formula Yinchenzhufu decoction (YCZFD). When administered as part of the traditional Yinchenzhufu (YCZFD) decoction, Atr was rapidly absorbed (*t*_max_ < 2 h) but eliminated more rapidly (*t*_1/2_ < 4 h). Interestingly, it had the highest systemic exposure (AUC_0−*t*_) among all detected constituents, and compared to Atr from *Atractylodes* extract, the form derived from YCZFD had a shorter half‐life but higher dose‐normalized *C*_max_ and AUC_0−*t*_, suggesting that other components of the decoction enhance its absorption while accelerating its clearance (Zan et al. 2022). The mechanism underlying this efficient absorption is confirmed by in vitro studies, which provide a cellular‐level explanation for the observed pharmacokinetic data. Mechanistic studies further confirm that Atr crosses the intestinal epithelium mainly by passive diffusion, with permeability coefficients (Papp values) that confirm its effective intestinal absorption (Wu et al. 2009). In summary, the pharmacokinetic profile of Atr indicates favorable bioavailability, characterized by efficient absorption, distribution, metabolism, and elimination. Its suitable half‐life further supports its potential for oral administration. The data collectively suggest that Atr's absorption is robust both as a pure compound and within a complex formula, though the herbal matrix can significantly alter its clearance kinetics, a critical consideration for its use in traditional medicine.

## 4 Conclusion and Perspectives

In this systematic review, guided by the PRISMA diagram, we demonstrated that Atr is a volatile sesquiterpenoid compound found in several medicinal plants, with the genus *Atractylodes* representing its main natural source. Its biosynthesis relies on two central terpenoid pathways, the MVA pathway and the MEP pathway, reflecting the metabolic flexibility of plants in producing this compound. Data collected in the literature highlight that Atr exerts multiple biological activities, including anti‐inflammatory, anticancer, neuroprotective, and gastroprotective effects, supported by various mechanistic studies. Furthermore, pharmacokinetic studies suggest that Atr exhibits efficient intestinal absorption, favorable bioavailability, and adequate metabolic stability, reinforcing its potential for therapeutic development. These findings support the idea that Atr could be considered an interesting candidate for pharmaceutical and cosmetic applications.

Nevertheless, the current body of evidence remains relatively limited, and several gaps persist, particularly regarding its pharmacodynamics, safety margins, and toxicity thresholds. To advance the field, further studies (especially well‐designed in vivo and clinical trials) are required to validate its efficacy and safety. Addressing these aspects will be crucial to fully assess Atr's potential and to translate preclinical findings into real‐world therapeutic applications.

## Author Contributions

**Hamza Elhrech** and **Oumayma Aguerd:** Data duration. **Abdelhakim Bouyahya:** Conceptualization. **Hamza Elhrech:** Formal analysis. **Waleed Al Abdulmonem:** Funding acquisition. **Taoufiq Benali, Waleed Al Abdulmonem**, and **Learn‐Han Lee:** Investigation. **Hamza Elhrech**, **Oumayma Aguerd**, **Meriem El Fessikh**, **Zouhair Essahli:** Methodology. **Abdelhakim Bouyahya:** Project administration. **Learn‐Han Lee**, **Imane Chamkhi**, and **Abdelhakim Bouyahya:** Validation. **Imane Chamkhi** and **Abdelhakim Bouyahya:** Supervision. **Learn‐Han Lee**, **Imane Chamkhi**, and **Abdelhakim Bouyahya:** Visualization. **Hamza Elhrech,** **Taoufiq Benali, Waleed Al Abdulmonem**, **Learn‐Han Lee, Imane Chamkhi**, and **Abdelhakim Bouyahya:** Writing – review and editing.

## Funding

This work was supported by the Deanship of Graduate Studies and Scientific Research at Qassim University for financial support (QU‐APC‐2026).

## Conflicts of Interest

The authors declare no conflicts of interest.

## Data Availability

The data that support the findings of this study are available from the corresponding author upon reasonable request.

## References

[fsn371488-bib-0001] Ahmed, S. , C. Zhan , Y. Yang , et al. 2016. “The Transcript Profile of a Traditional Chinese Medicine, *Atractylodes lancea*, Revealing Its Sesquiterpenoid Biosynthesis of the Major Active Components.” PLoS One 11, no. 3: e0151975. 10.1371/journal.pone.0151975. 26990438 PMC4798728

[fsn371488-bib-0002] Amorim, A. C. L. , C. K. F. Lima , A. M. C. Hovell , A. L. P. Miranda , and C. M. Rezende . 2009. “Antinociceptive and Hypothermic Evaluation of the Leaf Essential Oil and Isolated Terpenoids From *Eugenia uniflora* L. (Brazilian Pitanga).” Phytomedicine 16, no. 10: 923–928. 10.1016/j.phymed.2009.03.009. 19423309

[fsn371488-bib-0003] Burneo, J. I. , Á. Benítez , J. Calva , P. Velastegui , and V. Morocho . 2021. “Soil and Leaf Nutrients Drivers on the Chemical Composition of the Essential Oil of *Siparuna muricata* (Ruiz & Pav.) A. DC. From Ecuador.” Molecules 26, no. 10: 2949. 10.3390/molecules26102949. 34063513 PMC8155955

[fsn371488-bib-0004] Chen, L.‐G. , Y.‐S. Jan , P.‐W. Tsai , et al. 2016. “Anti‐Inflammatory and Antinociceptive Constituents of Atractylodes Japonica Koidzumi.” Journal of Agricultural and Food Chemistry 64, no. 11: 2254–2262. 10.1021/acs.jafc.5b05841. 26919689

[fsn371488-bib-0005] Cheng, Y. , T. Chen , X. Yang , J. Xue , and J. Chen . 2019. “Atractylon Induces Apoptosis and Suppresses Metastasis in Hepatic Cancer Cells and Inhibits Growth In Vivo.” Cancer Management and Research 11: 5883–5894. 10.2147/CMAR.S194795. 31388314 PMC6607983

[fsn371488-bib-0006] Cheng, Y. , J.‐Y. Mai , T.‐L. Hou , J. Ping , and J.‐J. Chen . 2016. “Antiviral Activities of Atractylon From *Atractylodis rhizoma* .” Molecular Medicine Reports 14, no. 4: 3704–3710. 10.3892/mmr.2016.5713. 27600871 PMC5042776

[fsn371488-bib-0007] Cheng, Y. , J. Ping , J. Chen , Y. Fu , H. Zhao , and J. Xue . 2022. “Molecular Mechanism of Atractylon in the Invasion and Migration of Hepatic Cancer Cells Based on High‐Throughput Sequencing.” Molecular Medicine Reports 25, no. 4: 1–13. 10.3892/mmr.2022.12628. 35119084 PMC8845028

[fsn371488-bib-0008] Ciccio, J. F. , C. Chaverri , and C. Diaz . 2009. “Volatile Compounds of *Nectandra salicina* (Lauraceae) From Costa Rica and Their Cytotoxic Activity on Cell Lines.” Química Nova 32, no. 2: 417–420. 10.1590/S0100-40422009000200028.

[fsn371488-bib-0009] da Thomas Silva, D. , C. Garrido Pinheiro , N. H. Bianchini , et al. 2018. “Microbiological Damage Influences the Content, Chemical Composition and the Antifungal Activity of Essential Oils in a Wild‐Growing Population of Ocotea Lancifolia (Schott) Mez.” Journal of Essential Oil Research 30, no. 4: 265–277. 10.1080/10412905.2018.1439409.

[fsn371488-bib-0010] de Santana Oliveira, M. , J. da Neves Cruz , W. da Almei Costa , et al. 2020. “Chemical Composition, Antimicrobial Properties of Siparuna Guianensis Essential Oil and a Molecular Docking and Dynamics Molecular Study of Its Major Chemical Constituent.” Molecules 25, no. 17: 3852. 10.3390/molecules25173852. 32854178 PMC7503653

[fsn371488-bib-0011] Gu, S. , L. Li , H. Huang , B. Wang , and T. Zhang . 2019. “Antitumor, Antiviral, and Anti‐Inflammatory Efficacy of Essential Oils From *Atractylodes macrocephala* Koidz. Produced With Different Processing Methods.” Molecules 24, no. 16: 2956. 10.3390/molecules24162956. 31443182 PMC6719198

[fsn371488-bib-0012] Han, N.‐R. , P.‐D. Moon , S.‐Y. Nam , et al. 2016. “Inhibitory Effects of Atractylone on Mast Cell‐Mediated Allergic Reactions.” Chemico‐Biological Interactions 258: 59–68. 10.1016/j.cbi.2016.08.015. 27553716

[fsn371488-bib-0013] Hung, N. H. , L. T. Huong , N. T. Chung , et al. 2020. “Callicarpa Species From Central Vietnam: Essential Oil Compositions and Mosquito Larvicidal Activities.” Plants 9, no. 1: 113. 10.3390/plants9010113. 31963227 PMC7020218

[fsn371488-bib-0014] Hwang, J.‐M. , T.‐H. Tseng , Y.‐S. Hsieh , F.‐P. Chou , C. J. Wang , and C.‐Y. Chu . 1996. “Inhibitory Effect of Atractylon on Tert‐Butyl Hydroperoxide Induced DNA Damage and Hepatic Toxicity in Rat Hepatocytes.” Archives of Toxicology 70, no. 10: 640–644. 10.1007/s002040050323. 8870957

[fsn371488-bib-0015] Iwasa, M. , T. Iwasaki , T. Ono , and M. Miyazawa . 2014. “Chemical Composition and Major Odor‐Active Compounds of Essential Oil From PINELLIA TUBER (Dried Rhizome of *Pinellia ternata* ) as Crude Drug.” Journal of Oleo Science 63, no. 2: 127–135. 10.5650/jos.ess13092. 24500103

[fsn371488-bib-0016] Kim, H.‐Y. , S.‐Y. Nam , S.‐Y. Hwang , H.‐M. Kim , and H.‐J. Jeong . 2016. “Atractylone, an Active Constituent of KMP6, Attenuates Allergic Inflammation on Allergic Rhinitis *In Vitro* and *In Vivo Models* .” Molecular Immunology 78: 121–132. 10.1016/j.molimm.2016.09.007. 27636508

[fsn371488-bib-0017] Lago, J. H. G. , E. D. Souza , B. Mariane , et al. 2011. “Chemical and Biological Evaluation of Essential Oils From Two Species of Myrtaceae—*Eugenia uniflora* L. and *Plinia trunciflora* (O. Berg) Kausel.” Molecules 16, no. 12: 9827–9837. 10.3390/molecules16129827. 22117172 PMC6264170

[fsn371488-bib-0018] Li, H. , F. Wang , Z. Zhou , et al. 2022. “Atractylon, a Novel Dopamine 2 Receptor Agonist, Ameliorates Parkinsonian Like Motor Dysfunctions in MPTP‐Induced Mice.” Neurotoxicology 89: 121–126. 10.1016/j.neuro.2022.01.010. 35104500

[fsn371488-bib-0019] Li, J. , F. Li , Y. Xu , et al. 2013. “Chemical Composition and Synergistic Antioxidant Activities of Essential Oils From *Atractylodes macrocephala* and *Astragalus membranaceus* .” Natural Product Communications 8, no. 9: 1934578X1300800934. 10.1177/1934578X1300800934. 24273876

[fsn371488-bib-0020] Li, L. , Y. Du , Y. Wang , N. He , B. Wang , and T. Zhang . 2022. “Atractylone Alleviates Ethanol‐Induced Gastric Ulcer in Rat With Altered Gut Microbiota and Metabolites.” Journal of Inflammation Research 15: 4709–4723. 10.2147/JIR.S372389. 35996682 PMC9392477

[fsn371488-bib-0021] Li, L. , Y. He , N. Wang , et al. 2023. “Atractylone in the *Atractylodes macrocephala* Rhizoma Essential Oil and Its Anti‐Inflammatory Activity.” Molecules 28, no. 21: 7340. 10.3390/molecules28217340. 37959758 PMC10648463

[fsn371488-bib-0022] Liu, H. , L. Kong , D. Cao , et al. 2024. “Efficacy and Mechanism of the Ermiao San Series of Formulas for Rheumatoid Arthritis Based on Chinmedomics Strategy.” Phytomedicine 132: 155903. 10.1016/j.phymed.2024.155903. 39047412

[fsn371488-bib-0023] Liu, Q. , S. Zhang , X. Yang , et al. 2016. “Differentiation of Essential Oils in *Atractylodes lancea* and *Atractylodes koreana* by Gas Chromatography With Mass Spectrometry.” Journal of Separation Science 39, no. 24: 4773–4780. 10.1002/jssc.201600574. 27790838

[fsn371488-bib-0024] Mao, J. , X. Wang , M. Yu , and C. Sun . 2022. “Effects of Atractylon on Proliferation and Apoptosis of Intestinal Cancer Cells Through PI3K/AKT/mTOR Signaling Pathway.” Cellular and Molecular Biology 68, no. 5: 153–160. 10.14715/cmb/2022.68.5.21. 36029491

[fsn371488-bib-0025] Martviset, P. , W. Chaijaroenkul , P. Muhamad , and K. Na‐Bangchang . 2018. “Bioactive Constituents Isolated From *Atractylodes lancea* (Thunb.) DC. Rhizome Exhibit Synergistic Effect Against Cholangiocarcinoma Cell.” Journal of Experimental Pharmacology 10: 59–64. 10.2147/JEP.S177032. 30498376 PMC6207387

[fsn371488-bib-0026] Miyazawa, M. , and J. Kawata . 2006. “Composition of the Essential Oil of Rootstock From *Cimicifuga simplex* .” Natural Product Research 20, no. 6: 542–547. 10.1080/14786410500182037. 16835085

[fsn371488-bib-0027] Pino, J. A. , C. E. Quijano‐Celis , and J. A. Rangel‐Mendoza . 2009. “Volatile Compounds of the Fruits of *Sipharuna thecaphora* (Poepp. et Endl.) A. DC.” Journal of Essential Oil Research 21, no. 4: 289–292. 10.1080/10412905.2009.9700173.

[fsn371488-bib-0028] Ren, Y. , W. Jiang , C. Luo , X. Zhang , and M. Huang . 2021. “The Promotive Effect of the Active Ingredients of *Atractylodes macrocephala* on Intestinal Epithelial Repair Through Activating Ca2+ Pathway.” Natural Product Communications 16, no. 11: 1934578X211040357. 10.1177/1934578X211040357.

[fsn371488-bib-0029] Resch, M. , A. Steigel , Z.‐l. Chen , and R. Bauer . 1998. “5‐Lipoxygenase and Cyclooxygenase‐1 Inhibitory Active Compounds From *Atractylodes lancea* .” Journal of Natural Products 61, no. 3: 347–350. 10.1021/np970430b. 9544564

[fsn371488-bib-0030] Ruan, Q. , J. Wang , C. Xiao , et al. 2021. “Differential Transcriptome Analysis of Genes Associated With the Rhizome Growth and Sesquiterpene Biosynthesis in *Atractylodes macrocephala* .” Industrial Crops and Products 173: 114141. 10.1016/j.indcrop.2021.114141.

[fsn371488-bib-0031] Sun, J. , H. Luo , Y. Jiang , L. Wang , C. Xiao , and L. Weng . 2022. “Influence of Nutrient (NPK) Factors on Growth, and Pharmacodynamic Component Biosynthesis of *Atractylodes chinensis*: An Insight on Acetyl‐CoA Carboxylase (ACC), 3‐Hydroxy‐3‐Methylglutaryl‐CoA Reductase (HMGR), and Farnesyl Pyrophosphate Synthase (FPPS) Signaling Responses.” Frontiers in Plant Science 13: 799201. 10.3389/fpls.2022.799201. 35371119 PMC8972053

[fsn371488-bib-0032] Sun, S. , J. Shi , X. Wang , et al. 2022. “Atractylon Inhibits the Tumorigenesis of Glioblastoma Through SIRT3 Signaling.” American Journal of Cancer Research 12, no. 5: 2310–2322. 35693089 PMC9185613

[fsn371488-bib-0033] Tian, C. , H. Liu , Q. Wang , et al. 2024. “Sanhan Huashi Formula and Its Bioactive Compounds Exert Antiviral and Anti‐Inflammatory Effects on COVID‐19.” Engineering 43: 159–172. 10.1016/j.eng.2024.07.007.

[fsn371488-bib-0034] Tian, M. , Q. Liu , Z. Yu , et al. 2019. “Attractylone Attenuates Sepsis‐Associated Encephalopathy and Cognitive Dysfunction by Inhibiting Microglial Activation and Neuroinflammation.” Journal of Cellular Biochemistry 120, no. 5: 7101–7108. 10.1002/jcb.27983. 30672013

[fsn371488-bib-0035] Tran, K. N. , N. P. K. Nguyen , L. T. H. Nguyen , H.‐M. Shin , and I.‐J. Yang . 2023. “Screening for Neuroprotective and Rapid Antidepressant‐Like Effects of 20 Essential Oils.” Biomedicine 11, no. 5: 1248. 10.3390/biomedicines11051248. PMC10215971 37238920

[fsn371488-bib-0036] Tsusaka, T. , B. Makino , R. Ohsawa , and H. Ezura . 2019. “Genetic and Environmental Factors Influencing the Contents of Essential Oil Compounds in *Atractylodes lancea* .” PLoS One 14, no. 5: e0217522. 10.1371/journal.pone.0217522. 31136627 PMC6538177

[fsn371488-bib-0037] Wang, C.‐C. , L.‐G. Chen , and L.‐L. Yang . 2002. “Cytotoxic Activity of Sesquiterpenoids From *Atractylodes ovata* on Leukemia Cell Lines.” Planta Medica 68: 204–208. 10.1055/s-2002-23144. 11914954

[fsn371488-bib-0038] Wang, K.‐T. , L.‐G. Chen , D.‐S. Chou , W.‐L. Liang , and C.‐C. Wang . 2011. “Anti‐Oxidative Abilities of Essential Oils From *Atractylodes ovata* Rhizome.” Evidence‐Based Complementary and Alternative Medicine 2011, no. 1: 204892. 10.1093/ecam/neq006. 21799672 PMC3135905

[fsn371488-bib-0039] Wang, K.‐T. , L.‐G. Chen , L.‐L. Yang , W.‐M. Ke , H.‐C. Chang , and C.‐C. Wan . 2007. “Analysis of the Sesquiterpenoids in Processed *Atractylodis rhizoma* .” Chemical and Pharmaceutical Bulletin 55, no. 1: 50–56. 10.1248/cpb.55.50. 17202701

[fsn371488-bib-0040] Wang, W. , Y. Jiang , B. Song , et al. 2023. “Discovery of Quality Markers in the Rhizome of *Atractylodes chinensis* Using GC–MS Fingerprint and Network Pharmacology.” Arabian Journal of Chemistry 16, no. 10: 105114. 10.1016/j.arabjc.2023.105114.

[fsn371488-bib-0042] Wu, Q. , and Y. Hu . 2020. “Integrated Network Pharmacology and Molecular Docking Strategy to Explore the Mechanism of Medicinal and Edible Astragali Radix‐Atractylodis Macrocephalae Rhizoma Acting on Pneumonia via Immunomodulation.” Journal of Food Biochemistry 44, no. 12: e13510. 10.1111/jfbc.13510. 33025599

[fsn371488-bib-0041] Wu, Q. , B. Zhao , X.‐W. Yang , et al. 2009. “Intestinal Permeability of Sesquiterpenes in the Caco‐2 Cell Monolayer Model.” Planta Medica 76: 319–324. 10.1055/s-0029-1186195. 19830652

[fsn371488-bib-0043] Wu, W. , S. Wang , J. Wu , B. He , B. Zhu , and L. Qin . 2021. “Influence of Tissue and Geographic Locality on Culturable Endophytic Bacteria of *Atractylodes macrocephala* .” Microbiology 167, no. 11: 001109. 10.1099/mic.0.001109. 34825886

[fsn371488-bib-0044] Yan, H. , Y. Sun , Y. Ma , et al. 2015. “Determination of Atractylon in Rat Plasma by a GC–MS Method and Its Application to a Pharmacokinetic Study.” Journal of Pharmaceutical Analysis 5, no. 5: 327–331. 10.1016/j.jpha.2015.03.002. 29403946 PMC5762239

[fsn371488-bib-0045] Yang, S. , J. Zhang , Y. Yan , et al. 2020. “Network Pharmacology‐Based Strategy to Investigate the Pharmacologic Mechanisms of *Atractylodes macrocephala* Koidz. for the Treatment of Chronic Gastritis.” Frontiers in Pharmacology 10: 1629. 10.3389/fphar.2019.01629. 32063848 PMC7000373

[fsn371488-bib-0046] Yao, Y. , G. Shen , J. Luo , et al. 2024. “Research Progress With Atractylone as an Antitumor Agent.” Molecules 29, no. 22: 5450. 10.3390/molecules29225450. 39598839 PMC11597220

[fsn371488-bib-0047] Yu, S. , K. Yasukawa , and M. Takido . 1994. “ *Atractylodis rhizoma* Extract and Its Component, Atractylon, Inhibit Tumor Promotion in Mouse Skin Two‐Stage Carcinogenesis.” Phytomedicine 1, no. 1: 55–58. 10.1016/S0944-7113(11)80023-1. 23195816

[fsn371488-bib-0048] Yuan, J. , W. Zhang , K. Sun , et al. 2019. “Comparative Transcriptomics and Proteomics of *Atractylodes lancea* in Response to Endophytic Fungus Gilmaniella sp. AL12 Reveals Regulation in Plant Metabolism.” Frontiers in Microbiology 10: 1208. 10.3389/fmicb.2019.01208. 31191508 PMC6546907

[fsn371488-bib-0049] Zan, B. , Y. Li , X. Sun , T. Wang , R. Shi , and Y. Ma . 2022. “Volatile Components in Yinchenzhufu Decoction and Their Pharmacokinetics after Oral Administration in Rats.” RSC Advances 12, no. 6: 3287–3299. 10.1039/D1RA08584K. 35425370 PMC8979343

[fsn371488-bib-0050] Zeng, H. , H. Gao , M. Zhang , et al. 2021. “Atractylon Treatment Attenuates Pulmonary Fibrosis via Regulation of the Mmu_circ_0000981/miR‐211‐5p/TGFBR2 Axis in an Ovalbumin‐Induced Asthma Mouse Model.” Inflammation 44, no. 5: 1856–1864. 10.1007/s10753-021-01463-6. 33855682

[fsn371488-bib-0051] Zhang, C. , H. Wang , C. Lyu , et al. 2023. “Authenticating the Geographic Origins of *Atractylodes lancea* Rhizome Chemotypes in China Through Metabolite Marker Identification.” Frontiers in Plant Science 14: 1237800. 10.3389/fpls.2023.1237800. 37841605 PMC10569125

[fsn371488-bib-0052] Zhang, W. , Q. Bai , G. Cui , et al. 2023. “Recent Progress and Ongoing Challenges in Rhizoma Atractylodis Research: Biogeography, Biosynthesis, Quality Formation and Control.” Medicinal Plant Biology 2, no. 1. 10.48130/MPB-2023-0019.

[fsn371488-bib-0053] Zhang, Z. , Y. Tian , X. Qiao , et al. 2025. “Integrated Analysis of Terpenoid Profiles and Full‐Length Transcriptome Reveals the Central Pathways of Sesquiterpene Biosynthesis in *Atractylodes chinensis* (DC.) Koidz.” International Journal of Molecular Sciences 26, no. 3: 1074. 10.3390/ijms26031074. 39940836 PMC11818032

[fsn371488-bib-0054] Zhuang, L.‐X. , Y. Liu , S.‐Y. Wang , et al. 2022. “Cytotoxic Sesquiterpenoids From *Atractylodes chinensis* (DC.) Koidz.” Chemistry & Biodiversity 19, no. 12: e202200812. 10.1002/cbdv.202200812. 36328982

[fsn371488-bib-0055] 杨雪丽 , 薛建华 , 陈天阳 , et al. 2021. “苍术酮对肝癌 HepG2 细胞活性、凋亡的影响及其相关机制.” Journal of Clinical Hepatology/Linchuang Gandanbing Zazhi | EBSCOhost 37, no. 11: 2589. 10.3969/j.issn.1001-5256.2021.11.020.

